# Health care workers hospitalized for COVID-19 in Liberia: who were they, and what were their outcomes?

**DOI:** 10.12688/f1000research.149673.1

**Published:** 2024-06-18

**Authors:** Darius B. Lehyen, Louis Ako-Egbe, Emmanuel Dwalu, Benjamin T. Vonhm, Pruthu Thekkur, Rony Zachariah, Luke Bawo

**Affiliations:** 1 Department of Infectious Diseases and Epidemiology, National Public Health Institute of Liberia, Monrovia, 1000, Liberia; 2 Health System Strengthening Department, World Health Organization, Country Office, Monrovia, 1000, Liberia; 3 Operational Research Department, Centre for Operational Research, International Union Against Tuberculosis and Lung Disease, Paris, 75001, France; 4 Implementation Research Department, UNICEF, UNDP, World Bank, WHO Special Programme for Research and Training in Tropical Diseases (TDR), Geneva, 1211, Switzerland; 5 Health Information, Monitoring & Evaluation and Research Department, Ministry of Health, Monrovia, 1000, Liberia

**Keywords:** SORT IT, Universal Health Coverage, Health Systems Strengthening, Infection Prevention Control, Pandemics, Operational Research, STROBE Guidelines, EpiCollect5

## Abstract

**Background:**

Sustaining a ‘fit-for-purpose’ health workforce requires a better understanding of the health care worker cadres that are affected during pandemics and their outcomes. In hospitalized health care workers with confirmed COVID-19 between March 2020 and May 2023 in Liberia, we determined the hospitalization and case fatality rates, type of health care worker cadres affected, their demographic and clinical characteristics and hospital exit outcomes.

**Methods:**

This was a cohort study using routine data extracted from hospitalization forms for health care workers in 24 designated COVID-19 treatment facilities in Liberia.

**Results:**

Of the 424 health care workers with COVID-19, hospitalization rates progressively declined between 2020 and 2023, with the highest rates in 2020 (24/1,000 health care workers) and 2021 (14/1,000 health care workers). Case fatality was 2% in both 2020 and 2021 with no deaths thereafter.

Among those hospitalized, the highest proportions were nursing cadres with 191(45%), physicians with 63 (15%) and laboratory technicians with 42 (10%). The most frequent reported site for COVID-19 infection was the health facility (326, 89%). COVID-19 vaccination coverage in health care workers was 20%. The majority (91%) of hospitalizations were for mild symptomatic infections. Even in referral centres (n-52), 18 (35%) were for mild infections.

Of the 424 who were hospitalized, 412 (97%) recovered, 9 (2%) died and 3 (1%) either left against medical advice or absconded. Of the nine deaths, none were vaccinated, seven had moderate-to-severe disease but were not referred to specialized COVID-19 treatment centers.

**Conclusions:**

The hospitalized health care workers for COVID-19 were predominantly clinical and laboratory personnel who were mostly unvaccinated, and health facilities were hot-spots for contracting infections. The triage and referral system was weak with unnecessary hospitalization of mild infections. This study provides useful insights for outbreak preparedness including priority vaccination and improving health care worker safety in Liberia.

## Introduction

An umbrella review of 13 systematic reviews from 1,230 studies including 1,040,336 COVID-19 confirmed cases revealed a global infection rate of 14.5% among health care workers, and an overall case fatality of nine deaths per 1,000 infections. ^1^ These rates could have been accentuated by health-related challenges among one-third of the health care workers such as poor sleep quality, mental health issues, work-related stress and skin injuries associated with use of personal protective equipment. ^2^ There was also limited access to COVID-19 vaccines. ^3^

In the African region, the World Health Organisation (WHO) estimated that over 10,000 health care workers were infected with COVID-19. Liberia reported a total of 8,090 confirmed COVID-19 cases and 404 healthcare worker infections to WHO, ranking it among the top three with the highest country-specific health care worker infection rates in the region. ^4^

Prior to the COVID-19 pandemic, the 2014-2016 Ebola outbreak in Liberia took a devastating toll on health care workers with 372 infections and 184 Ebola-related deaths which reduced the health workforce by 8.1%. ^5^ The country has one of the lowest core health care worker density ratios in the world with 12.8 core health care workers per 10,000 population (WHO minimum = 44.5 per 10,000 habitants). For example, in the USA, this figure stands at 82 per 10,000 ( WHO - health-workforce). Thus, preventing health care worker deaths is a top priority for sustaining the Liberian health system and achieving Universal Health Coverage. ^6^

The dire health care worker situation in Liberia underpins the importance of health care worker safety and the need to effectively monitor health care worker infections using standardized data surveillance and clinical management tools. During outbreaks, such data collection tools are often not available, and countries may be “caught unprepared”. Thus, there is the need to establish timely standardized and regular surveillance, case management and reporting systems. Data validation during outbreaks is also a challenge as the health system gets rapidly overwhelmed and busy health care workers end up often unable to devote time for data validation. The situation was no different in Liberia which, in 2020, introduced a paper-based data collection form for health care workers hospitalized with COVID-19. To date, there has been no formal audit of the completeness and usefulness of this data in informing outbreak management.

An operational research priority in Liberia is to better understand the health care worker cadres that were affected by COVID-19, their clinical course, and outcomes. The existing data collection forms provide an opportunity to analyze such information using an operational research lens. Such information could contribute towards sustaining a ‘fit-for-purpose’ health workforce and improving the monitoring system in preparation for future outbreaks. ^7^ A PUBMED search revealed no studies from Liberia (or neighbouring countries) that assessed the quality of COVID-19 data for health care workers hospitalized to health facilities and their outcomes.

We thus aimed to describe the characteristics of hospitalized health care workers with COVID-19 in Liberia and their outcomes. In all hospitalized health care workers with confirmed COVID-19 in Liberia between March 2020 and May 2023, our specific objectives were to determine a) the COVID-19 hospitalization and case fatality rates by year, b) the type of health care worker cadres affected along with their demographic and clinical characteristics, c) the medical interventions administered and d) the health facility exit outcomes. We also assessed the proportion of missing and unknown data.

## Methods

### Study design

Cohort study using routine country-wide health facility data.

### General setting

Liberia is a country on the West African coast, bordered by Sierra Leone to the northwest, Guinea to the north, the Ivory Coast to the east, and the Atlantic Ocean to the south. It has a population of around 5 million and is divided into 15 counties (with Montserrado county being the most populated and hosting the capital city of Monrovia) and 98 districts.

The health system is tiered and includes a primary level (primary health clinics), a secondary level (health center and county/regional hospitals) and a tertiary level (national specialized hospitals). The first case of COVID-19 in Liberia was confirmed on 16 ^th^ March 2020, and by July 2023 there had been a total of 8,090 confirmed cases. ^5^ Vaccination of health care workers started in late 2021. By 30 April 2023, complete COVID-19 vaccination coverage in the general population was 81% and was similar in health care workers ( WHO - COVID-19 vaccination drive).

### Specific setting

Screening, diagnosis, and management of COVID-19 in health care workers

COVID-19 was managed according to the national guideline. ^8^ In brief, all health care workers with clinical signs (e.g., fever, cough, sore throat) were tested for the COVID-19 virus (SARS-CoV-2) using nasopharyngeal samples. SARS-CoV-2 was confirmed using Reverse Transcriptase Polymerase Chain Reaction (RT-PCR). ^9^ Those with moderate to severe clinical symptoms were screened for common comorbidities (e.g., HIV, diabetes mellitus, asthma, and hypertension). Clinical management was based on four principles: early identification, early isolation and quarantine, early diagnosis, and early treatment.

In terms of clinical management, patients were graded according to WHO criteria as: mild, moderate, severe and critical ( Table 1). ^8^ Asymptomatic patients with mild disease were monitored at home and those with symptomatic disease (irrespective of severity) were hospitalized on clinical discretion of the medical team. Symptomatic patients were managed at one of the COVID-19 treatment centers. Those with severe and critical disease were to be administered oxygen by nasal cannulae or face mask, and if needed, hospitalized to an intensive care unit (ICU). Mechanical respiratory support (where available) and management of complications (pneumonia, sepsis, acute respiratory distress, and other life-threatening conditions) were to be done in ICUs.

**Table 1.** Classification of COVID-19 severity among health care workers hospitalized for confirmed COVID-19 in Liberia (March 2020 – May 2023).

Severity grade	Clinical criteria
Mild	Symptomatic without pneumonia
Moderate	Symptomatic with pneumonia SpO _2_ ≥ 92%
Severe	Oxygen saturation SpO _2_<92% on room air Signs of severe pneumonia Signs of severe respiratory distress: • RR>28-32bpm • Cyanosis • Chest in-drawing
Critical	Requires life sustaining treatment (e.g. mechanical ventilation) Acute Respiratory Distress Syndrome Sepsis Septic shock

SpO _2_=Saturation percentage of oxygen; RR=Respiratory Rate; bpm=breaths per minute.

COVID-19 facility exit outcomes were standardized as recovered and discharged, died, abandoned (patient left without notice) and left-against-medical-advice.

### Study population and period

The study population included all hospitalized health care workers with confirmed COVID-19 from March 2020 to May 2023 in Liberia. The study was conducted between July 2023 and March 2024.

### Study sites

There were 962 functional health facilities in the country of which 24 were designated as COVID-19 treatment facilities and located in fourteen counties. Twenty-two of these facilities were COVID-19 treatment units (first level) and two were specialized centers with an intensive care unit (referral level). The study sites were the 24 designated COVID-19 treatment facilities.

### Data collection and validation

Individual patient information from the various COVID-19 treatment units and centres were entered on data forms by the attending clinicians. The county case management officers in collaboration with the county surveillance team collated this information which was centralized at the National Public Health Institute and the Ministry of Health.

All data in the national COVID-19 database was reviewed by the principal investigator and a line list of health care workers hospitalized for COVID-19 between March 2020 and May 2023 was created. Thereafter, the principal investigator visited all treatment facilities and traced the individual patient cards for cross-validation of data in the line-list. The central Incident Management Team provided oversight and then entered all data into an EpiCollect5 mobile cloud-based application for further validation and analysis.

### Data variables and sources of data

Data variables included patient number, admitting COVID-treatment facility, health care worker type, demographic and clinical characteristics, co-morbidities, vaccination status, medical interventions, complications, and facility exit outcomes. The sources of data included the COVID-19 national health care worker database, individual patient cards in COVID-19 treatment facilities and the national database of human resources for health.

### Statistical analysis

**Figure 1.**
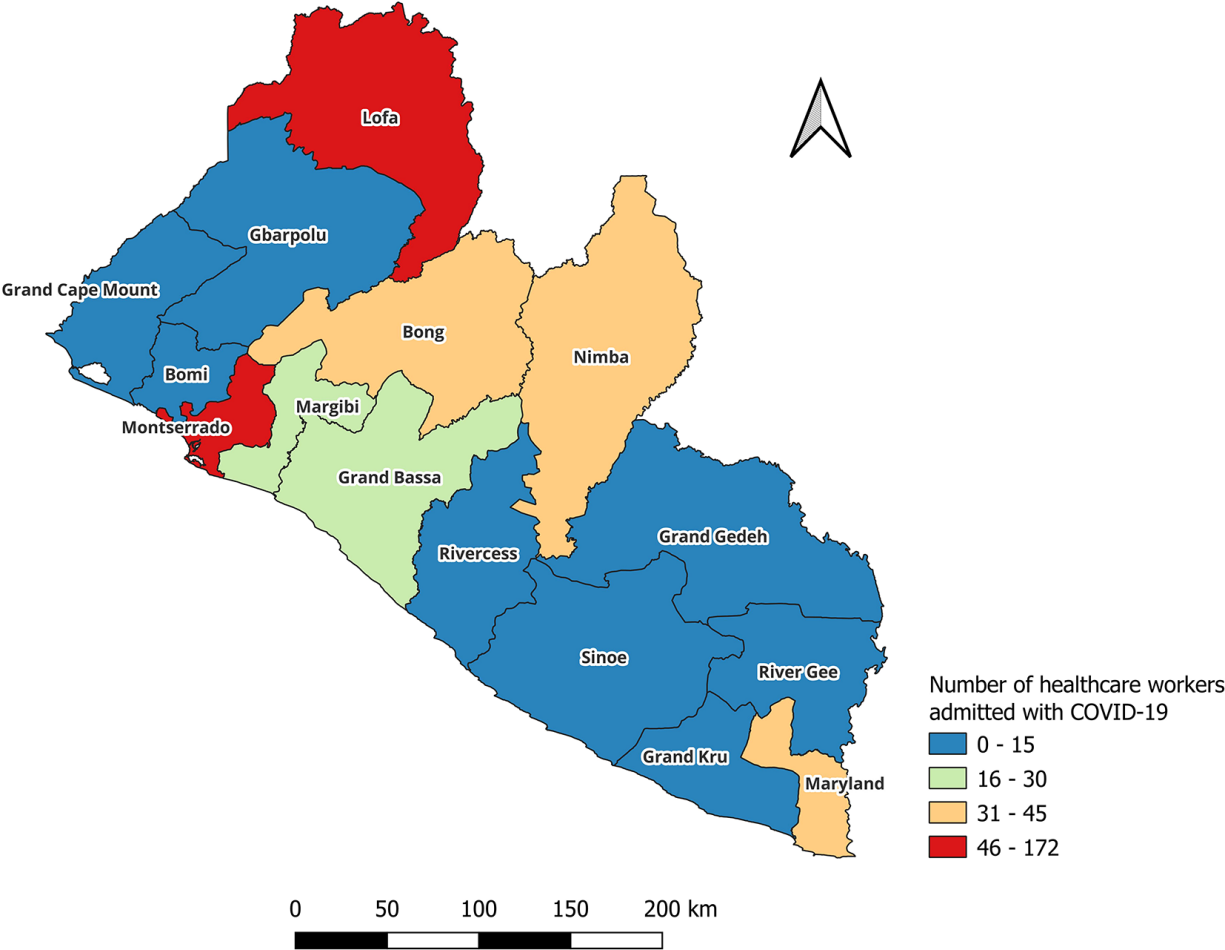
Countrywide geographic distribution of health care workers hospitalized for COVID-19 in Liberia (March 2020-May 2023).

Data was analyzed using ( Stata software version 16) (College Station, TX, USA: Stata Corp LLC. Free alternatives to Stata are RStudio or JASP). Countrywide geographic distribution of health care workers infected with COVID-19 was presented graphically per county using QGIS software 3.36.2 ( Figure 1).

The demographic characteristics, clinical characteristics, vaccination status and hospital exit outcomes were summarized using frequencies and percentages. Hospitalization rates for COVID-19 among health care workers were calculated by standardizing the numbers of health care workers hospitalized by year with the total number of health care workers registered in that year and expressed by 1,000 health care workers.

### Ethics approval

Permission for using the data was obtained from the Ministry of Health of Liberia. National Ethics approval was obtained from the Liberian National Ethics Review Board (Number 23-09-391, dated 27 ^th^ September 2023) and international approval was received from the Union Ethics Advisory Group of the Center for Operational Research of the International Union against Tuberculosis and Lung Disease, Paris, France (EAG number 18/23, dated 8 ^th^ September 2023). As we used secondary health facility data, the issue of informed consent did not apply.

## Results

### Number of hospitalized health care workers with COVID-19 and rates per 1000 (2020-2023)

Of the 424 health care workers hospitalized with COVID-19 in Liberia, the highest number was in 2020 (247 cases) followed by 2021 (149 cases). Hospitalizations showed a linear declining trend from 2020 to 2023 ( Table 2, chi-square for linear trend=321, P<0.001).

**Table 2.** Number of health care workers, health care workers confirmed with COVID-19 and hospitalization rates in Liberia from 2020 to 2023.

Year	Total health care workers (a)	Health care workers confirmed with COVID-19 (b)	Hospitalization rate ^1^ (b/a*1,000)	Number deaths/case fatality (%)
2020	**10,302**	**247**	**24**	**6 (2%)**
2021	**10,093**	**149**	**14**	**3 (2%)**
2022	**9,706**	**18**	**2**	**0**
2023	**9,802**	**10**	**1**	**0**
**Total**		**424**		

Chi-Square for linear trend = 321, P<0.001.

Similarly, the rate of hospitalization declined between 2020 and 2023 with the highest rates in 2020 (24/1,000 health care workers) and 2021 (14/1,000 health care workers). Case fatality was 2% in both 2020 and 2021. There were no deaths in 2022 and 2023.

Figure 1 shows countrywide geographic distribution of health care workers infected with COVID-19 graphically per county. The highest number of hospitalizations were in Montserrado and Lofa counties.

### Health care worker cadres and their demographic and clinical characteristics

Table 3 shows the type of health cadres hospitalized and their characteristics. Among those hospitalized, the highest proportions were nursing cadres (nurses, midwives, and nursing aids) with 191 (45%) hospitalized, followed by physicians (doctors and physician assistants) with 63 (15%), and laboratory technicians with 42 (10%). The most frequent reported site for COVID-19 exposure (presumed source of infection) was the health facility (326, 89%). At the time of hospitalization, COVID-19 vaccination coverage in health care workers was 20%. The majority (91%) of hospitalizations were for mild symptomatic infections. Even in referral centres (n-52), 18 (35%) were for mild infections.

**Table 3.** Health care worker cadres, demographic and clinical characteristics of those with confirmed COVID-19 in Liberia (March 2020-May 2023), N=424.

Characteristics	n	(%) ^$^
Health care worker		
Nurses/Midwives/Nursing aid	191	(45)
Doctors/Physician Assistant	65	(15)
Laboratory technician	42	(10)
Hospital Administrative staff	17	(4)
Environmental Health Technician	12	(3)
Cleaners	11	(3)
Community Health Volunteer	10	(2)
Others *	28	(7)
Not recorded	48	(11)
Severity		
Mild	387	(91)
Moderate	28	(7)
Severe	6	(1)
Unknown	2	(<1)
Not recorded	1	(<1)
Admitting facility		
COVID treatment Unit	372	(88)
COVID treatment Centre	52	(12)
Age (years)		
≤20	5	(1)
21-40	221	(52)
41-60	173	(41)
≥61	25	(6)
Sex		
Male	237	(56)
Female	187	(44)
Exposed to person with similar illness		
Yes	368	(87)
No	30	(7)
Unknown	26	(6)
Suspected place of exposure ^#^ (N=368)		
Health facilities	326	(89)
Home	10	(3)
Other	26	(7)
Not recorded	6	(1)
Vaccination status		
Yes	86	(20)
No	298	(70)
Unknown	39	(9)
Not recorded	1	(<1)
Symptoms presented with ^@^		
Fever > 38°C/history of fever	236	(56)
Running nose	140	(33)
Cough	213	(50)
Shortness of breath	53	(12)
No symptom recorded	75	(18)
Co-morbidities		
Hypertension	8	(2)
Diabetes mellitus	4	(1)
Obesity	1	(<1)
Asthma	1	(<1)
HIV	1	(<1)
Hepatitis	1	(<1)

^$^ Column percentage.

* Other Health care workers includes vaccinators, drivers, hospital security staff, pharmacists and dispensers.

# Only among those who reported to have been exposed to a person with similar illness.

^@^ Multiple symptoms are possible. Thus, total does not add up to 424.

Between one and ten percent of clinical variables related to demographics and clinical features had either missing information and/or unknown information.

### Medical interventions offered and complications

Table 4 shows the medical interventions and recorded complications. There were three individuals who received oxygen with oxygen concentrators. Five patients had life-threatening sepsis as a complication but all five were managed in first level-facilities (treatment units). There was one to three percent of missing information in variables related to medical interventions and complications.

**Table 4.** Medical interventions and clinical complications among health care workers hospitalized for confirmed COVID-19 in Liberia (March 2020-May 2023), N=424.

Characteristics	n	(%)
***Medical interventions***		
Oxygen alone		
Yes	3	(1)
No	421	(99)
Admitted in ICU		
Yes	1	(0.5)
No	409	(96)
Unknown	13	(3)
Unrecorded	1	(0.5)
Mechanical ventilation provided		
Yes	0	(0)
No	417	(98)
Unknown	3	(1)
Unrecorded	4	(1)
Extra Membrane Corporal Oxygenation		
Yes	0	(0)
No	416	(98)
Unknown	0	(0)
Unrecorded	8	(2)
***Complications***		
Pneumonia confirmed through X-rays		
Yes	0	(0)
No	414	(98)
Unknown	1	(0)
Unrecorded	9	(2)
Acute Respiratory Distress Syndrome		
Yes	1	(0)
No	411	(97)
Unknown	10	(2)
Unrecorded	2	(0)
Other life-threatening condition (Sepsis)		
Yes	5	(1)
No	406	(96)
Unrecorded	13	(3)

ICU=intensive care unit.

### Health facility exit outcomes

Of 424 health care workers hospitalized, 412 (97%) recovered and were discharged, 9 (2%) died and 3 (1%) either left against medical advice or absconded ( Table 5). Of the nine who died, none were vaccinated, seven had moderate-to-severe disease and all were hospitalized in non-specialized COVID-19 treatment units. In two deaths, disease severity was not recorded.

**Table 5.** Health facility-exit outcomes among health care workers hospitalized for COVID-19 in Liberia (March 2020-May 2023).

Outcomes	N	(%)
Total exits	424	
Recovered & discharged	412	(97)
Died	9	(2)
Left against medical advice	2	(0.5)
Abandoned	1	(0.5)

## Discussion

This first country-wide study from Liberia on health care worker hospitalizations for COVID-19 showed that clinical and laboratory personnel were the most affected, the main source of infection was the health facility and only 20% of health care workers were vaccinated. The majority (91%) of all hospitalizations were for mild symptomatic infections and this applied as well to the 35% of hospitalizations in specialized centres. Among those who died with moderate to severe infection, none had benefited from hospitalization in specialized centres.

The study findings are important as they are a clarion call for improving health care worker safety, strengthening infection prevention and control, and offering health care workers priority access to vaccination. In the early part of the pandemic, when vaccines were in short supply and/or equity issues were in play, ^10^ vaccinating health care workers should have been a top-level priority to sustain a ‘fit for purpose’ health workforce. This strategy is akin to “treating the pilots in a plane flying at 30,000 feet before attending to passengers”. However, Liberia like most other African countries did not have sufficient access to COVID-19 vaccines. ^11^ This study also highlights the importance of early institution of triage and referral systems to ensure that individuals with moderate to critical infections and most in need of hospitalized care, are adequately catered for during an outbreak.

The study strengths are that the research subject was a national operational research priority and thus relevant, and all health facilities in the country were included making it likely that the study was representative of the situation on the ground. We also ensured that data were cross-validated with individual patient cards at all study sites, and we adhered to the STROBE (Strengthening the Reporting of Observational Studies in Epidemiology) guidelines. ^12^

One of the study limitations is that we had missing and/or unknown variables (one to ten percent) in the patient cards, highlighting the need for enhanced oversight for improving the quality of data by the national and county incident management teams. The fact that these teams have already taken corrective steps with health facilities shows the useful synergy developed between operational research and the strengthening of the current monitoring and evaluation system. Another limitation is that we did not know the exact reasons for the observed decline in numbers of hospitalized cases by year and whether this was related to increasing vaccination coverage over time and/or changes in hospitalization practices or the virulence of the circulating SARS-CoV-2 virus strains.

This study has a number of policy and practice implications. First, clinical and laboratory staff constituted 70% of all hospitalizations and their predominant source of infection was the health facility. This highlights that health facilities were ‘hot spots’ for the acquisition and transmission of infection. The way forward is to ensure regular IPC assessments in all health facilities ^13^ and ensure the availability of on-site ‘safety stocks’ of Personal Protective Equipment (PPE) material as part of the preparedness strategy for future outbreaks. Furthermore, the fact that there were nine health care worker deaths from COVID-19 compounds the already low health care worker density in Liberia and this may have contributed to disruptions in routine health service delivery.

Second, the fact that the majority of hospitalizations (91%), including a considerable proportion in specialized centres, were for mild symptomatic infections is concerning. It implies that COVID-19 treatment sites largely served as ‘isolation facilities’ rather than treatment centres. From a health system perspective, this is disadvantageous for two reasons: a) available beds get unnecessarily filled and those genuinely meriting hospitalized care may risk being turned away, and b) large numbers of individuals with mild infections hospitalized in health facilities increases the risk of disease transmission to both health care workers and the community at large.

Clogging up the health system will also have a crippling effect resulting in those with moderate to severe disease not being promptly identified and referred to higher level health facilities. This seems to have been the case since nine health care workers who died should have been referred to specialized centres but this did not happen. Five individuals identified with complicated sepsis were also not referred to specialized treatment centres. Such shortcomings in the triage and referral system should be corrected for future outbreaks and public health emergencies.

Third, during outbreaks, there is need for data and information in ‘real-time’ to inform and enable decision makers to institute prompt actions that can uphold the capability and accountability of health systems. ^14^ A survey conducted by the WHO in 2022 showed that 92% of all countries were still facing COVID-19 related disruptions which affected all health service delivery platforms. ^15^ In Liberia, as of July 2021, utilization of essential maternal and reproductive health services was lower than pre-pandemic trends. Similarly, there were substantial disruptions in outpatient services (down by 17%), family planning consultations (down by 36%), post-natal care first visit (down by 21%), ante natal care first visit (down by 19%). ^16^ ‘Real-time intelligence’ would help steer the health system out of such trouble as has been demonstrated in some African countries. ^17^^,^^18^ The use of mobile phone applications (such as EpiCollect5) and cloud-based data storage can provide a useful adjunct to emergency responses and should be explored in Liberia and neighbouring countries. ^17^^,^^18^

## Conclusions

In conclusion, this country-wide study from Liberia showed that hospitalized health care workers for COVID-19 were predominantly clinical and laboratory personnel who were mostly unvaccinated, and health facilities were hot-spots for contracting infections. The triage and referral system was weak with unnecessary hospitalization of mild infections. This study provides useful insights for outbreak preparedness including priority vaccination and improving health care worker safety in Liberia.

## Data Availability

Requests to access these data should be sent to the corresponding author who will liaise with the Department of Infectious Diseases and Epidemiology (DIDE) at the National Public Health Institute of Liberia. All requests for access to the dataset will need to be submitted to a data committee of the DIDE.
