# Supplementary material for: Health care workers hospitalized for COVID-19 in Liberia: who were they, and what were their outcomes?
Source: F1000Res. 2024 Nov 14;13:656. Originally published 2024 Jun 18. [Version 2] doi: 10.12688/f1000research.149673.2 (PMC11574328; doi:10.12688/f1000research.149673.2)
Supplement: Peer review history

# Reviewer report: Reviewer response for version 2

**DOI:** 10.5256/f1000research.174181.r340612

**Published:** 2024-11-18

**Authors:** Helena C Maltezou

**Affiliations:** 1 National Public Health Organization, Athens, Greece

I went throughout the paper and I accept it in its current version.

Is the work clearly and accurately presented and does it cite the current literature?

No

If applicable, is the statistical analysis and its interpretation appropriate?

Yes

Are all the source data underlying the results available to ensure full reproducibility?

No source data required

Is the study design appropriate and is the work technically sound?

Yes

Are the conclusions drawn adequately supported by the results?

Yes

Are sufficient details of methods and analysis provided to allow replication by others?

Yes

Reviewer Expertise:

NA

I confirm that I have read this submission and believe that I have an appropriate level of expertise to confirm that it is of an acceptable scientific standard.

# Reviewer report: Reviewer response for version 1

**DOI:** 10.5256/f1000research.164163.r328861

**Published:** 2024-10-26

**Authors:** Helena C Maltezou

**Affiliations:** 1 National Public Health Organization, Athens, Greece

This is an interesting study. Please find below my comments:

1. Abstract: please provide p-values.
2. It is not clear why HCP were mostly not vaccinated. Were HCP prioritized for COVID-19 vaccination in Liberia? Please clarify. 
3. Discussion: The authors should compare their findings with those reported by other researchers.
4. Please move the strengths and limitations paragraph before the conclusion (last) paragraph. 

Is the work clearly and accurately presented and does it cite the current literature?

No

If applicable, is the statistical analysis and its interpretation appropriate?

Yes

Are all the source data underlying the results available to ensure full reproducibility?

No source data required

Is the study design appropriate and is the work technically sound?

Yes

Are the conclusions drawn adequately supported by the results?

Yes

Are sufficient details of methods and analysis provided to allow replication by others?

Yes

Reviewer Expertise:

Epidemiology, healthcare workers occupational risk, vaccination, COVID-19

I confirm that I have read this submission and believe that I have an appropriate level of expertise to confirm that it is of an acceptable scientific standard, however I have significant reservations, as outlined above.

# Response

**Published:** 2024-10-28

**Authors:** Darius B. Lehyen

Thanks so very much to the reviewer for highlighting the following key points on my submission

1. Abstract: P-value had been provided in the abstract section of the article
2. It is not clear why HCP were mostly not vaccinated. Were HCP prioritized for COVID-19 vaccination in Liberia? Please clarify. **Author response: **HCP were not vaccinated because in the early part of the pandemic, vaccines were in short supply and equity issues were in play. Offering health care workers priority access to COVID-19 vaccination was an issue from the onset of the pandemic in Liberia and this is mentioned in the article.
3. Discussion: The authors should compare their findings with those reported by other researchers. **Author response: **The findings of this article were compared with other researchers findings in the last paragraph in discussion.
4. **Author response: **The strengths and Limitations were moved to the last paragraph before the conclusion of the article.

# Reviewer report: Reviewer response for version 1

**DOI:** 10.5256/f1000research.164163.r316429

**Published:** 2024-08-26

**Authors:** Kenneth Nugent

**Affiliations:** 1 Department of Internal Medicine, Texas Tech University Health Sciences Center, Lubbock, Texas, USA

1. These authors have analyzed the healthcare workers in Liberia who had confirmed COVID-19 infection between March 2020 and May 2023. The study extracted routine data from hospitalization forms in 24 designated COVID-19 treatment facilities. In general, they provide a complete overview of these workers and their characteristics.
2. The following results seem important. None of the healthcare workers who died had been vaccinated. Seven of the nine who died had moderate to severe disease but were not referred to specialized treatment centers. Many patients with mild disease were hospitalized, possibly resulting in an unnecessary use of resources.
3. This project provides an outline which other healthcare facilities and/or organizations could use to analyze COVID-19 infections. In addition, it should provide the healthcare authorities in Liberia information to help them plan for the next epidemic.

Is the work clearly and accurately presented and does it cite the current literature?

Yes

If applicable, is the statistical analysis and its interpretation appropriate?

Yes

Are all the source data underlying the results available to ensure full reproducibility?

Yes

Is the study design appropriate and is the work technically sound?

Yes

Are the conclusions drawn adequately supported by the results?

Yes

Are sufficient details of methods and analysis provided to allow replication by others?

Yes

Reviewer Expertise:

Critical care medicine

I confirm that I have read this submission and believe that I have an appropriate level of expertise to confirm that it is of an acceptable scientific standard.

# Response

**Published:** 2024-08-26

**Authors:** Darius B. Lehyen

Thanks so kindly to the reviewer for highlighting the following key points on my submission

 1. That the work was clearly and accurately presented and cited the current literature.

 2. That the study design was appropriate and the work is technically sound.

 3. That there are sufficient details of methods and analysis provided to allow replication by others.

 4. That the statistical analysis and its interpretation is appropriate.

 5. That all the source data underlying the results are available to ensure full reproducibility and 

 6. That the conclusions drawn are adequately supported by the results

**I authorize that I have read the reviewer response to my submission and I believe that he has a suitable level of expertise to confirm that my article is of an acceptable scientific standard.**
